# Bis(cyclohexylammonium) 2,2′-disulfanediyldibenzoate

**DOI:** 10.1107/S1600536810054012

**Published:** 2011-01-12

**Authors:** Xinting Wei, Jing Li, Handong Yin

**Affiliations:** a College of Chemistry and Chemical Engineering, Liaocheng University, Shandong 252059, People’s Republic of China

## Abstract

In the title molecular salt, 2C_6_H_14_N^+^·C_14_H_8_O_4_S_2_^2−^, the complete dianion is generated by crystallographic twofold symmetry and a twisted conformation is found [the C—S—S—C torsion angle is 87.13 (2)° and the dihedral angle between the rings is 83.4 (2)°]. In the crystal, inter­molecular N—H⋯O hydrogen bonds link the cations and anions.

## Related literature

For the design and synthesis of novel coordination architectures, see: Sato *et al.* (1996 ▶); Yaghi *et al.* (1998 ▶). 
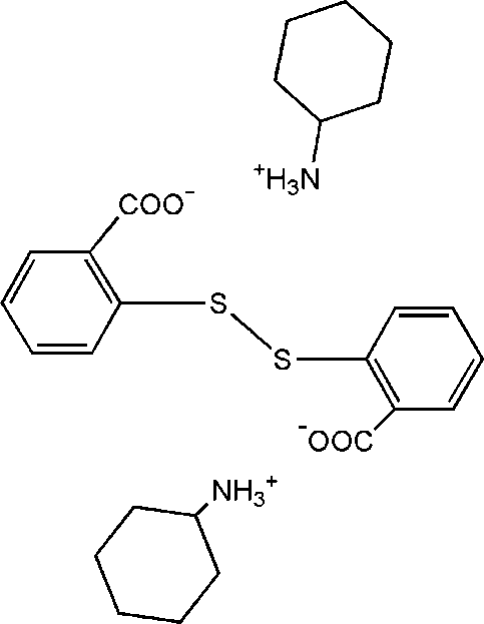


## Experimental

### 

#### Crystal data

2C_6_H_14_N^+^·C_14_H_8_O_4_S_2_^2−^
*M* *_r_* = 504.69
Tetragonal, 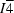

*a* = 11.6411 (15) Å
*c* = 20.105 (3) Å
*V* = 2724.6 (6) Å^3^
*Z* = 4
Mo *K*α radiation
μ = 0.23 mm^−1^
*T* = 298 K
0.48 × 0.46 × 0.42 mm

#### Data collection

Bruker SMART diffractometer
Absorption correction: multi-scan (*SADABS*; Sheldrick, 1996 ▶) *T*_min_ = 0.898, *T*_max_ = 0.910
5632 measured reflections
2394 independent reflections
1398 reflections with *I* > 2σ(*I*)
*R*_int_ = 0.036

#### Refinement

*R*[*F* ^2^ > 2σ(*F* ^2^)] = 0.045
*wR*(*F* ^2^) = 0.124
*S* = 1.05
2394 reflections
155 parameters
H-atom parameters constrained
Δρ_max_ = 0.17 e Å^−3^
Δρ_min_ = −0.17 e Å^−3^
Absolute structure: Flack (1983 ▶), 1153 Friedel pairs
Flack parameter: −0.07 (12)

### 

Data collection: *SMART* (Siemens, 1996 ▶); cell refinement: *SAINT* (Siemens, 1996 ▶); data reduction: *SAINT*; program(s) used to solve structure: *SHELXS97* (Sheldrick, 2008 ▶); program(s) used to refine structure: *SHELXL97* (Sheldrick, 2008 ▶); molecular graphics: *SHELXTL* (Sheldrick, 2008 ▶); software used to prepare material for publication: *SHELXTL*.

## Supplementary Material

Crystal structure: contains datablocks I, global. DOI: 10.1107/S1600536810054012/bx2336sup1.cif

Structure factors: contains datablocks I. DOI: 10.1107/S1600536810054012/bx2336Isup2.hkl

Additional supplementary materials:  crystallographic information; 3D view; checkCIF report

## Figures and Tables

**Table 1:** Hydrogen-bond geometry (Å, °)

*D*—H⋯*A*	*D*—H	H⋯*A*	*D*⋯*A*	*D*—H⋯*A*
N1—H1*C*⋯O1	0.89	1.91	2.785 (5)	167
N1—H1*A*⋯O1^i^	0.89	1.96	2.841 (5)	172
N1—H1*B*⋯O2^ii^	0.89	1.84	2.723 (5)	175

Symmetry codes: (i) 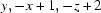; (ii) 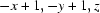.

**Fig. 1.**
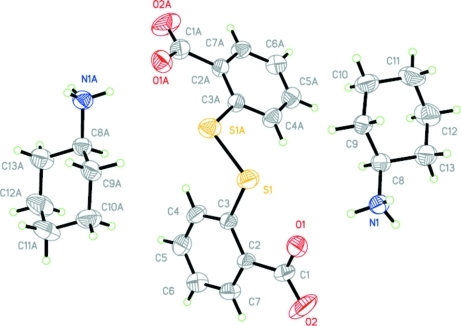
The molecular structure of the compound, showing 50% probability displacement ellipsoids. H atoms have been omitted for clarity. (#1 - x + 1,-y + 2,z)

**Fig. 2.**
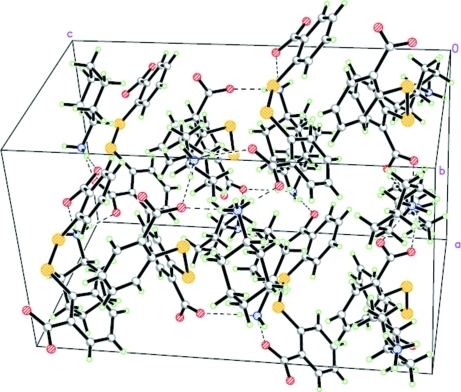
The crystal packing, linked by N—H···O hydrogen bonds. (#2 y,-x + 1,-z + 2 #3 - x + 1,-y + 1,z)

## supplementary crystallographic information

### Comment

The design and synthesis of novel coordination architectures has resulted in a great number of research efforts, due not only to their intriguing structural topologies, but also to their unexpected properties as functional materials (Sato *et al.*, 1996; Yaghi *et al.*, 1998). The main strategy popularly used in this area is the building–block approach. 2,2'–Dithiodibenzoic acid is a good choice in the design of novel coordination architectures, since its four coordination sites are likely to engage in coordination to metal ions. We report here the crystal structure, of new salt of 2,2-dithiodisalicylate with cyclohexylammonium cation. The title compound, (I) is composed of 2,2-dithiodisalicylate and cyclohexylammonium ions, in a ratio of 1:2; the asymmetric unit consists of one-half molecule of the 2,2-dithiodisalicylate and one cyclohexylammonium cation. A twofold axis of symmetry passes through the centre of the S—S bond. A twisted conformation is found for the anion [the C—S—S—C torsion angle is 87.13 (2)° and the dihedral angle between the rings is 83.4 (2)°]. There are two N—H···O intermolecular and one intramolecular hydrogen bonds, (Fig. 2 and Table 2).

### Experimental

The reaction was carried out under nitrogen atmosphere. 2,2'–Dithiodibenzoic acid (0.5 mmol) was dissolved in 10 ml methanol, and a solution of cyclohexylamine (1.0 mmol) in 20 ml toluene was added dropwise under intense agitation. The mixture was placed in air at room temperature. Suitable for X-ray analysis were obtained by slow evaporation of acetone solution over a period of two weeks. Analysis, calculated for [(C_14_H_8_O_4_S_2_)^2-^.2(C_6_H_14_N)^+^ (Mr = 504.69): C 61.87, N 5.55, H 7.19%; found: C 61.82, N 5.50, H 7.15%.

### Refinement

The H atoms were positioned geometrically with aromatic C—H distances of 0.93 Å, and refined as riding on their parent atoms, with *U*_iso_(H) = 1.2 *U*_eq_(C). The H atoms were positioned geometrically with cyclohexylamine C—H distances of 0.97 Å and refined as riding on their parent atoms, with *U*_iso_(H) = 1.5 *U*_eq_(C). The N—H distance is 0.89 Å with *U*_iso_(H) = 1.2 *U*_eq_(N).

### Crystal data

**Table:**

2C_6_H_14_N^+^·C_14_H_8_O_4_S_2_^2^^−^	*D*_x_ = 1.230 Mg m^−^^3^
*M**_r_* = 504.69	Mo *K*α radiation, λ = 0.71073 Å
Tetragonal, *I*4	Cell parameters from 1336 reflections
Hall symbol: I -4	θ = 3.2–18.6°
*a* = 11.6411 (15) Å	µ = 0.23 mm^−^^1^
*c* = 20.105 (3) Å	*T* = 298 K
*V* = 2724.6 (6) Å^3^	Block, colourless
*Z* = 4	0.48 × 0.46 × 0.42 mm
*F*(000) = 1080	

### Data collection

**Table:**

Bruker SMART diffractometer	2394 independent reflections
Radiation source: fine-focus sealed tube	1398 reflections with *I* > 2σ(*I*)
graphite	*R*_int_ = 0.036
phi and ω scans	θ_max_ = 25.0°, θ_min_ = 3.2°
Absorption correction: multi-scan (*SADABS*; Sheldrick, 1996)	*h* = −13→7
*T*_min_ = 0.898, *T*_max_ = 0.910	*k* = −12→13
5632 measured reflections	*l* = −23→22

### Refinement

**Table:**

Refinement on *F*^2^	Secondary atom site location: difference Fourier map
Least-squares matrix: full	Hydrogen site location: inferred from neighbouring sites
*R*[*F*^2^ > 2σ(*F*^2^)] = 0.045	H-atom parameters constrained
*wR*(*F*^2^) = 0.124	*w* = 1/[σ^2^(*F*_o_^2^) + (0.0468*P*)^2^ + 1.0167*P*] where *P* = (*F*_o_^2^ + 2*F*_c_^2^)/3
*S* = 1.05	(Δ/σ)_max_ < 0.001
2394 reflections	Δρ_max_ = 0.17 e Å^−^^3^
155 parameters	Δρ_min_ = −0.17 e Å^−^^3^
0 restraints	Absolute structure: Flack (1983), 1153 Friedel pairs
Primary atom site location: structure-invariant direct methods	Flack parameter: −0.07 (12)

### Fractional atomic coordinates and isotropic or equivalent isotropic displacement parameters (Å^2^)

**Table:**

	*x*	*y*	*z*	*U*_iso_*/*U*_eq_	
N1	0.6618 (3)	0.6089 (3)	0.93400 (16)	0.0705 (10)	
H1A	0.6773	0.5904	0.9760	0.106*	
H1B	0.6574	0.5454	0.9096	0.106*	
H1C	0.5951	0.6462	0.9322	0.106*	
O1	0.4378 (3)	0.6925 (2)	0.92848 (17)	0.0819 (9)	
O2	0.3371 (4)	0.5845 (3)	0.8579 (2)	0.1379 (17)	
S1	0.48337 (10)	0.91347 (10)	0.91518 (6)	0.0769 (4)	
C1	0.3688 (5)	0.6790 (4)	0.8808 (3)	0.0799 (14)	
C2	0.3236 (3)	0.7842 (3)	0.8463 (2)	0.0575 (11)	
C3	0.3708 (4)	0.8932 (3)	0.85641 (19)	0.0572 (10)	
C4	0.3259 (4)	0.9841 (4)	0.8194 (2)	0.0729 (13)	
H4	0.3587	1.0566	0.8235	0.088*	
C5	0.2343 (5)	0.9691 (4)	0.7772 (2)	0.0786 (13)	
H5	0.2045	1.0318	0.7543	0.094*	
C6	0.1871 (4)	0.8638 (5)	0.7687 (2)	0.0757 (13)	
H6	0.1244	0.8542	0.7406	0.091*	
C7	0.2327 (4)	0.7705 (4)	0.8022 (2)	0.0691 (12)	
H7	0.2022	0.6977	0.7950	0.083*	
C8	0.7554 (4)	0.6845 (3)	0.9074 (2)	0.0643 (11)	
H8	0.7267	0.7221	0.8671	0.077*	
C9	0.7850 (4)	0.7765 (4)	0.9571 (2)	0.0739 (13)	
H9A	0.8072	0.7412	0.9989	0.089*	
H9B	0.7182	0.8242	0.9652	0.089*	
C10	0.8830 (4)	0.8502 (4)	0.9313 (3)	0.0922 (16)	
H10A	0.8579	0.8914	0.8919	0.111*	
H10B	0.9040	0.9063	0.9648	0.111*	
C11	0.9852 (4)	0.7788 (5)	0.9145 (3)	0.1032 (17)	
H11A	1.0138	0.7418	0.9544	0.124*	
H11B	1.0457	0.8275	0.8970	0.124*	
C12	0.9542 (5)	0.6881 (5)	0.8633 (3)	0.110 (2)	
H12A	1.0208	0.6405	0.8542	0.132*	
H12B	0.9314	0.7250	0.8221	0.132*	
C13	0.8565 (4)	0.6133 (5)	0.8887 (3)	0.0919 (16)	
H13A	0.8344	0.5591	0.8544	0.110*	
H13B	0.8823	0.5700	0.9271	0.110*	

### Atomic displacement parameters (Å^2^)

**Table:**

	*U*^11^	*U*^22^	*U*^33^	*U*^12^	*U*^13^	*U*^23^
N1	0.062 (2)	0.059 (2)	0.091 (3)	−0.0040 (17)	0.0074 (19)	−0.0104 (19)
O1	0.070 (2)	0.076 (2)	0.100 (2)	0.0034 (17)	0.002 (2)	0.0247 (19)
O2	0.186 (4)	0.051 (2)	0.177 (4)	−0.026 (3)	−0.053 (3)	0.031 (3)
S1	0.0775 (8)	0.0692 (8)	0.0839 (7)	−0.0112 (7)	−0.0063 (7)	0.0131 (7)
C1	0.075 (3)	0.055 (3)	0.110 (4)	−0.011 (3)	0.018 (3)	0.016 (3)
C2	0.056 (3)	0.049 (2)	0.067 (3)	−0.004 (2)	0.019 (2)	0.007 (2)
C3	0.057 (3)	0.052 (3)	0.062 (2)	0.000 (2)	0.016 (2)	0.005 (2)
C4	0.086 (3)	0.050 (3)	0.083 (3)	−0.005 (2)	0.008 (3)	0.010 (2)
C5	0.091 (4)	0.073 (4)	0.072 (3)	0.006 (3)	−0.005 (3)	0.007 (3)
C6	0.084 (4)	0.082 (4)	0.061 (3)	−0.008 (3)	0.005 (2)	−0.001 (3)
C7	0.071 (3)	0.066 (3)	0.070 (3)	−0.015 (2)	0.013 (3)	−0.005 (2)
C8	0.062 (3)	0.063 (3)	0.068 (3)	−0.005 (2)	0.000 (2)	−0.003 (2)
C9	0.059 (3)	0.063 (3)	0.099 (3)	0.003 (2)	0.006 (2)	−0.025 (3)
C10	0.088 (4)	0.073 (3)	0.115 (4)	−0.023 (3)	0.010 (3)	−0.014 (3)
C11	0.064 (3)	0.122 (5)	0.123 (4)	−0.025 (3)	0.012 (3)	−0.024 (4)
C12	0.078 (4)	0.131 (5)	0.121 (4)	−0.011 (4)	0.027 (3)	−0.034 (4)
C13	0.074 (3)	0.084 (3)	0.118 (4)	−0.003 (3)	0.019 (3)	−0.035 (3)

### Geometric parameters (Å, °)

**Table:**

N1—C8	1.499 (5)	C7—H7	0.9300
N1—H1A	0.8900	C8—C13	1.488 (6)
N1—H1B	0.8900	C8—C9	1.504 (6)
N1—H1C	0.8900	C8—H8	0.9800
O1—C1	1.260 (5)	C9—C10	1.520 (6)
O2—C1	1.248 (6)	C9—H9A	0.9700
S1—C3	1.780 (4)	C9—H9B	0.9700
S1—S1^i^	2.051 (2)	C10—C11	1.490 (7)
C1—C2	1.503 (6)	C10—H10A	0.9700
C2—C7	1.390 (6)	C10—H10B	0.9700
C2—C3	1.397 (5)	C11—C12	1.518 (7)
C3—C4	1.396 (5)	C11—H11A	0.9700
C4—C5	1.374 (6)	C11—H11B	0.9700
C4—H4	0.9300	C12—C13	1.521 (7)
C5—C6	1.355 (6)	C12—H12A	0.9700
C5—H5	0.9300	C12—H12B	0.9700
C6—C7	1.383 (6)	C13—H13A	0.9700
C6—H6	0.9300	C13—H13B	0.9700
			
C8—N1—H1A	109.5	N1—C8—H8	108.0
C8—N1—H1B	109.5	C9—C8—H8	108.0
H1A—N1—H1B	109.5	C8—C9—C10	110.3 (4)
C8—N1—H1C	109.5	C8—C9—H9A	109.6
H1A—N1—H1C	109.5	C10—C9—H9A	109.6
H1B—N1—H1C	109.5	C8—C9—H9B	109.6
C3—S1—S1^i^	105.63 (14)	C10—C9—H9B	109.6
O2—C1—O1	125.4 (5)	H9A—C9—H9B	108.1
O2—C1—C2	116.3 (5)	C11—C10—C9	111.2 (4)
O1—C1—C2	118.2 (4)	C11—C10—H10A	109.4
C7—C2—C3	119.8 (4)	C9—C10—H10A	109.4
C7—C2—C1	117.9 (4)	C11—C10—H10B	109.4
C3—C2—C1	122.3 (4)	C9—C10—H10B	109.4
C4—C3—C2	117.6 (4)	H10A—C10—H10B	108.0
C4—C3—S1	121.9 (3)	C10—C11—C12	110.6 (4)
C2—C3—S1	120.5 (3)	C10—C11—H11A	109.5
C5—C4—C3	121.6 (4)	C12—C11—H11A	109.5
C5—C4—H4	119.2	C10—C11—H11B	109.5
C3—C4—H4	119.2	C12—C11—H11B	109.5
C6—C5—C4	120.5 (5)	H11A—C11—H11B	108.1
C6—C5—H5	119.7	C11—C12—C13	110.4 (4)
C4—C5—H5	119.7	C11—C12—H12A	109.6
C5—C6—C7	119.6 (5)	C13—C12—H12A	109.6
C5—C6—H6	120.2	C11—C12—H12B	109.6
C7—C6—H6	120.2	C13—C12—H12B	109.6
C6—C7—C2	120.8 (4)	H12A—C12—H12B	108.1
C6—C7—H7	119.6	C8—C13—C12	111.0 (4)
C2—C7—H7	119.6	C8—C13—H13A	109.4
C13—C8—N1	109.8 (4)	C12—C13—H13A	109.4
C13—C8—C9	112.5 (4)	C8—C13—H13B	109.4
N1—C8—C9	110.3 (3)	C12—C13—H13B	109.4
C13—C8—H8	108.0	H13A—C13—H13B	108.0
			
O2—C1—C2—C7	13.8 (6)	C4—C5—C6—C7	−0.9 (7)
O1—C1—C2—C7	−168.7 (4)	C5—C6—C7—C2	2.4 (7)
O2—C1—C2—C3	−165.3 (5)	C3—C2—C7—C6	−0.9 (6)
O1—C1—C2—C3	12.3 (6)	C1—C2—C7—C6	180.0 (4)
C7—C2—C3—C4	−2.0 (6)	C13—C8—C9—C10	54.8 (6)
C1—C2—C3—C4	177.0 (4)	N1—C8—C9—C10	177.8 (4)
C7—C2—C3—S1	177.4 (3)	C8—C9—C10—C11	−55.9 (6)
C1—C2—C3—S1	−3.5 (5)	C9—C10—C11—C12	57.7 (6)
S1^i^—S1—C3—C4	2.2 (4)	C10—C11—C12—C13	−57.1 (7)
S1^i^—S1—C3—C2	−177.2 (3)	N1—C8—C13—C12	−178.4 (4)
C2—C3—C4—C5	3.6 (6)	C9—C8—C13—C12	−55.1 (6)
S1—C3—C4—C5	−175.9 (4)	C11—C12—C13—C8	55.5 (6)
C3—C4—C5—C6	−2.1 (7)		

Symmetry codes: (i) −*x*+1, −*y*+2, *z*.

### Hydrogen-bond geometry (Å, °)

**Table:**

*D*—H···*A*	*D*—H	H···*A*	*D*···*A*	*D*—H···*A*
N1—H1C···O1	0.89	1.91	2.785 (5)	167
N1—H1A···O1^ii^	0.89	1.96	2.841 (5)	172
N1—H1B···O2^iii^	0.89	1.84	2.723 (5)	175

Symmetry codes: (ii) *y*, −*x*+1, −*z*+2; (iii) −*x*+1, −*y*+1, *z*.
